# Developing a reliable and valid competency model for assistant dentists in China

**DOI:** 10.1186/s12909-021-02986-0

**Published:** 2021-10-29

**Authors:** Xiaomei Hong, Yueping Li, Zhong Chen, Yingzhen Lai, Qinyue Dai, Hao Liang

**Affiliations:** 1 Health Research Institute of Fujian Medical University, 350108 Fuzhou, Fujian China; 2 Department of Stomatology, Xiamen Medical College, 361023 Xiamen, Fujian China; 3 Engineering Research Center of Fujian University for Stomatological Biomaterials, Xiamen Medical College, 361023 Xiamen, Fujian China

**Keywords:** Health services research, assistant dentists, Competency model, Characteristics of oral professionals, Oral health

## Abstract

**Background:**

The shortage of dentists is one of the various medical-resource problems found around the world. More and more countries are improving the provision of oral services by training dental therapists and hygienists. In China, they are called assistant dentists, and they supplement dental services, but thus far, no research has been conducted on the competence of this group.

**Objective:**

The purpose of this study was to establish a competency model for Chinese assistant dentists. This model can provide a more scientific basis for the education, training, and evaluation of these professionals, as well as provide a reference for the capacity of dental therapists in various countries.

**Methods:**

We established a system of competency characteristics using theoretical analysis and focus group discussions, after which we established an initial competency model by consulting a Delphi panel of 29 experts. Finally, we collected data from 1389 assistant dentists from 14 provinces in China, and the reliability and validity of the model were confirmed by factor analysis of this data.

**Results:**

After three rounds of Delphi panels, the competency model came to include seven ability levels and 50 indicators. In exploratory-factor analysis, three indicators were eliminated, and the questionnaire could explain 68.41 % of total variance. In confirmatory-factor analysis, the established model and data fit well (goodness-of-fit index [GFI] = 0.914, root mean square error of approximation [RMSEA] = 0.047). The results showed that the entire model has good reliability and validity.

**Conclusions:**

Our competency model for dental assistants in China includes seven elements. This is consistent with the current health situation in China, and it has distinct Chinese characteristics. Some of our findings, like those reported in other countries with dental therapists, offer ideas for other developing countries.

## Introduction

Oral diseases are considered a major global-health burden and public health challenge [1–4]. Although the present situation has received attention from the World Health Organization (WHO), the prevalence of oral diseases continues to rise in most low- and middle-income countries (LMICs) and regions [5, 6]. A major reason for this is poor access to community-based oral-healthcare services in these places [7]. To address this problem, some of these countries are starting to create levels of qualification for jobs supplementing and assisting dentists. For example, New Zealand started to introduce dental therapists (formerly called “school dental nurses”) into the workforce as early as 1921 [7]. Dental therapists must complete a 3-year degree in oral health to provide basic preventive and restorative dental care to children in school dental services (SDS) [8, 9]. In Australia, Canada, and Malaysia, dental therapists have provided the vast majority of oral-healthcare services to children in school-based clinics [10, 11]. Since the beginning of the 21st century, dental therapists and hygienists have comprised a new type of professional auxiliary dentist around the globe, and a trend of these professionals integrating into oral-health therapy plans has become noticeable [11]. As in most countries, poor access to health services exists in China, particularly in rural and other economically underdeveloped areas. However, instead of employing dental therapists or hygienists to fill this gap, China has created the clinical post of assistant dentist to deal with the shortage of dentists.

Generally, Chinese dentists are required to attend a college, medical institute, or university for 5 years to obtain a bachelor’s degree in medicine. After graduation, under the guidance of a practicing dentist, graduates who have completed a one-year probation at a medical institution may take the National Dental Licensing Examination of China for qualification. Only after passing the exam can one obtain a dental license to practice medicine independently in China. Assistant dentists are the unique product of China’s national conditions and is set up to supplement primary medical staff. There are some differences between dentists and assistant dentists in the training and assessment process. Dental assistants require only 3 years of specialized dental courses at a vocational college (without a bachelor’s degree). One year after graduation, college graduates take the National Assistant Dental Licensing Examination for qualification and obtain an assistant-dentist license certificate. Finally, after further training, the assistant dentist passes a rigorous exam and has the chance to become a dentist.

The job duties of assistant dentists vary by region and by the requirements and conditions of the practice organization. This scope varies according to the distribution of medical resources, as is the case with dental-health aides in the United States (US)[12]. Whether they are dental therapists/hygienists or assistant dentists, they are all Professionals Complementary to Dentistry (PCDs). The quality and responsibility of these professionals is an important factor in the quality of medical services. The training and education of PCDs has become one of the most debated topics in medical human resources in China, so it is necessary to re-examine the range of their professional skills and duties [13, 14]. In the early 21st century, medical education gradually changed from problem based to competency based [15], and the evaluation and training of medical staff have changed accordingly. To date, research on the competence of medical personnel has tended to focus on doctors and nurses rather than on professionals in supplementary roles [11, 16]. The American Dental Education Association (ADEA), National Dental Examining Board of Canada (NDEB), Association for Dental Education in Europe (ADEE), and Australian Dental Association (ADA) have put forward skill requirements for dentists [16–20]. Although extensive studies have been conducted on the orientation, cultivation and job satisfaction of dental therapists, no single study has been able to address the competence requirements for this group of PCDs. In particular, there is no competency model for this group in China, and it is not clear what skills assistant dentists need.

The specific objective of this study was to construct a competency model for assistant dentists taking into account China’s national conditions. The model can provide a scientific basis for the education, training, and assessment of assistant dentists, as well as provide a reference for PCDs’ competence in various countries. This is the first paper to put forth a competency model for assistant dentists in China. We also discuss how to increase the introduction of assistant dentists to provide reference data that countries, especially low-income countries, can use to solve the dental shortage problem. Because our thesis does not engage with specific competencies, throughout this paper, the term “competency” will refer to general competencies.

## Methods

### Design and procedure

**Fig. 1:**
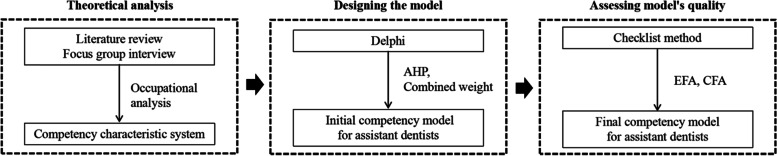
Technological Flow of Research

Our study was conducted from October 2018 to January 2020, and all questionnaires were supported by the National Medical Examination Center (NMEC) of China. The construction of the general competency model for assistant dentists combined the advantages of qualitative and quantitative analysis and was divided into three steps. Figure 1 shows the technological flow of our research.

System of competency characteristics.

First, after reviewing the literature and studying dental-competency models from various countries such as the US, New Zealand, Australia, and Canada, we referred to the framework of the competency model for doctors and dentists in China, which we obtained from research conducted at China Medical University (Shenyang, China) and Capital Medical University (Beijing, China) [21, 22]. We used focus group interviews to collect and build a pool of competencies that assistant dentists should have. We organized five focus group discussions involving different types of professionals or lay people: assistant dentists, dentists, heads of dental clinics, educational experts, and patients. The number of participants in each interview was 8–10. The selection of experts was agreed by NMEC and the research team, and the relevant personnel from each region were invited by stratified target sampling according to seven regions in China. The inclusion criteria of interviewees were clear language expression, good communication skills, and willingness to participate in the study. The expert interviewees should be representative and authoritative. Representativeness means that experts can represent a group of people, and authoritativeness means that experts have rich experience or higher academic attainments in the field of nursing personnel training and management. On this basis, all kinds of interviewees also need to meet the following criteria: education experts should have associate professors or above titles; heads of dental clinics should have at least 5 years of working experience; additionally dentists should have intermediate or above professional titles, more than 10 years of working experience, and 2 years or more of working experience as assistant dentists; the patient should have been treated by an assistant dental practitioner within one year. The interview lasted for 60 to 120 min until information saturation and no new information emerged. Based on eight dimensions obtained from the literature, participants conducted position analysis of the duties, knowledge, skills, personality traits, and other characteristics that are desirable in assistant dentists. The core questions were, “What does an assistant dentist have to do in their job?” and, “What capabilities should assistant dentists have?” After integration and optimization, the final results of the discussion formed a preliminary competency characteristic system with 8 first- and 58 s-level abilities. The eight first-level abilities are (1) clinical skills and medical care, (2) health promotion, (3) medical knowledge and lifelong learning, (4) communication and interpersonal skills, (5) teamwork, (6) research ability, (7) information management ability and (8) professional values.

Initial competency model for assistant dentists.

Second, based on the abovementioned competency characteristic system, we created a Delphi questionnaire on the competency of assistant dentists. After considering factors such as professional qualifications, which may differ by region, and acting on recommendations from the National Medical Examination, we selected 30 experts for a Delphi panel. They were from Peking University, Shanghai Jiaotong University, Sichuan University, Wuhan University and other universities and their affiliated oral hospitals; teachers from 15 higher vocational colleges; and senior teachers in clinics. There were 58 items on the Delphi questionnaire. In each round, panelists were asked to grade competencies in three dimensions—importance, feasibility and sensitivity—that should be developed in medical practice, and to provide arguments for their ratings, until they all agreed on indicators. After questionnaires were returned, we screened and modified the indicators in combination with the experts’ revised opinions, and then the next round of consultation was conducted. This continued until expert opinions were unanimous. In the final round, we compared every pair of factors and ranked all indicators from the most to the least important so that we could compute their weight. We used email to communicate with experts and to execute all phases of the Delphi study. So far, this work has established a preliminary theoretical model of competence for assistant dentists.

Final competency model for assistant dentists.

Third, we created a checklist based on comprehensive consideration of the initial competency model. Our method was to list relevant problems by the characteristics of the research objects (assistant dentists), arrange them into a checklist and then discuss them one by one to find creative solutions. In order to avoid sequence bias, we randomly sorted the items in the initial competency model established in the Delphi panels, compiled the checklist and judged the importance of each item on a Likert scale. We purposefully selected 1500 assistant dentists from different institutions to participate in this checklist survey. The data collected from the questionnaire were tested for reliability and validity to verify the scientific nature and reliability of the competency model and obtain a final competency model for assistant dentists.

### Questionnaires

We designed a questionnaire to ascertain participants’ opinions on the competency of assistant dentists. The competency indices of the initial model obtained via Delphi panel were randomly sorted and their importance evaluated. Participants were then asked to rate them on a 5-point Likert scale, with 1–5 indicating “very unimportant,” “not important,” “average,” “important” and “very important,” respectively. The checklist questionnaire consisted of 50 items, such as, “How important is making a reasonable diagnosis and treatment plan to the performance of assistant dentists’ work?” and, “How important is the ability to operate and recognize the root tip to the performance of the assistant dentist?” A higher score indicated that assistant dentists considered the item more important to competence.

### Participants

There are seven geographical regions in China. From each, we chose 1–3 provinces, for a total of 14 provinces. Regions (provinces) were as follows: North China (Tianjin), Northeast China (Jilin and Heilongjiang), East China (Fujian, Anhui and Shandong), Central China (Hubei and Henan), South China (Guangdong), Southwest China (Chongqing, Guizhou and Yunnan) and Northwest China (Qinghai and Shanxi). Assistant dentists at the national level were our main objects of study, and we used a stratified proportion sampling method and 1500 samples. The number of samples was calculated based on the average number of candidates who are take the oral assistant-physician examinations held in the province in the past 6 years (provided by the NMEC of China). 10 % of candidates responded.

Because this survey involved a large number of questionnaires and spanned a broad geography with scattered populations, the quality of the questionnaire data might not have been well controlled. Therefore, we coded the districts and participating institutions uniformly before conducting the survey. The final questionnaire was coded as “district code + institution code + questionnaire number,” which played a great role in the follow-up statistical questionnaire.

### Statistical analysis

In the Delphi study, to assess the consistency of different experts’ opinions, we used Kendall’s coefficient as the parameter. It can reflect whether expert opinion is deeply divided in the evaluation of competency indicators. To calculate weight, we used the analytic hierarchy process (AHP) and the combined-weight method. Weight indicates the relative importance of the index, which can provide a reference value for application of the competency model.

Next, we tested the reliability and validity of the competency model using the inspection survey data. Reliability and validity are the main properties measured by measurement instruments [23, 24], which are usually employed during construction and evaluation, so that the design quality of the questionnaire can be assessed. We used Cronbach’s alpha, a coefficient commonly used to indicate the reliability of scales, to test the internal consistency of reliability of the entire questionnaire and of seven first-level indicators. Construct validity is based on the construction of hypotheses, which are tested to support effectiveness [25]. Factor analysis is a widely used technique to verify construct validity. Therefore, we used exploratory-factor analysis (EFA) and a structural-equation model (confirmatory-factor analysis [CFA]) to test our model’s validity [26]. If the structure of an assumed competency model is consistent with EFA and CFA results, the model is reasonable and effective. After completing reliability and validity testing, we constructed a final model of competence in one’s post for assistant physicians. CFA results were reported with different statistical coefficients of goodness of fit [27]. We used EpiData software v3.1 (The EpiData Association, Odense, Denmark) to input all data from these experiments; data were entered twice to ensure accuracy. Data management and analysis were performed using Microsoft Excel (Microsoft Corp., Redmond, WA, USA) and SPSS software version 18.0 (IBM Corp., Armonk, NY, USA). *P* < 0.05 was considered statistically significant.

## Results

### Delphi panels

We conducted three rounds of Delphi panels. A total of 30 experts participated, although 1 of them dropped out, leaving 29. Of the experts who completed our survey, 82.76 % had master’s degrees or higher, 68.97 % had senior professional titles, and 96.55 % had >10 years’ work experience. The effective questionnaire contained 29 cases, with an effective return rate of 96.67 % over the three rounds of e-questionnaires, indicating that most panelists were interested in and willing to participate in this research. The expert authority coefficient in each round of consultation was 0.774–0.909, higher than 0.7, indicating that Delphi content validity was satisfactory.

After summarizing and analyzing experts’ opinions in the first round, we revised some indicators. For example, research ability, one of the first-level indices, has been deleted. Experts believed that although research ability is easy to assess and is very important to physicians, it is not required for dentists at the assistant level. At the end of the first round, we deleted one first-level and eight second-level indicators, and we modified the expression, among which two second-level indicators were deleted along with scientific research ability, and six indicators disappeared after merging (Table 1). In the second round of consultation, we fed the results of the first round back to the experts. In this round, panelists were asked to score the revised indicators once again. This time the experts reached greater consensus on the revised indices. In the third and final round, pairwise comparisons were made, and indices were sorted and ordered by the weights of all factors. We used AHP and the combined-weight method to calculate the weights of the initial competency indicators.

**Table 1:** Secondary indicators deleted and merged after the first round of Delphi

Pre-merger (deleted) indicators	Indicators after consolidation
Consciousness of scientific research	/
Literature retrieval and reading skills	
Good doctor–patient communication	Good doctor–patient relationship
Consider the patient	
Patients’ right to know	Communicate the diagnosis and treatment plan
Inform the treatment plan	
Make plans through cooperation	Coordinating member relationships
Collaborative completion therapy	
Consciousness of self-protection	Avoid occupational exposure
Self-protection ability	
Controlling healthcare costs	Avoid medical waste
Rational use of resources	
Use inspection system	Use health information systems
Use dental imaging system	

### Exploratory-factor analysis

From July 2019 to November 2019, a total of 1500 questionnaires were sent out, and 1389 were recovered. After we excluded invalid questionnaires (with incomplete data), we had a total of 1286 valid questionnaires, with an effective recovery rate of 85.73 %. In general, sample size was the ratio of questions to participants, 1:10 to 1:15. There were 55 questions on the checklist questionnaire. Accordingly, the number of recovered questionnaires satisfied the requirements of subsequent analysis. We randomly divided the data into two groups, and we used different samples in the EFA and CFA to avoid self-verification with the same sample.

The results indicated that the internal-consistency coefficient of the whole scale and sub-scale was >0.7. The research showed that a coefficient of Cronbach’s alpha ≥0.7 was good for reliability [28]. Furthermore, Kaiser–Meyer–Olkin (KMO) value was 0.971, and Bartlett’s test value was χ^2^ = 29350.13 (df = 1225; *P* < 0.001. KMO test value being >0.7 and the statistical significance of Bartlett’s test value indicated that the sample was sufficient for factor analysis [29]. Hence, both reliability and validity met the needs of factor analysis.

We used principal-component analysis (PCA) and varimax rotation with Kaiser normalization to extract 50 items. Following the Kaiser criterion (i.e., eigenvalues of all extracted factors were >1, and the load of all factors on a factor was >0.45) [30], we extracted seven common factors and excluded some items. Three secondary indicators (item20, item21, and item32) were ultimately deleted because the load was <0.45. Clinical skills and medical care include the diagnosis and treatment techniques and medical services provided by assistant dentists to patients in the actual clinical operation process, with a total of 15 items. Medical knowledge and lifelong learning include the ability of continuous self-learning and the mastery of relevant professional knowledge that assistant dentists need to have in the process of work, with a total of four items. Communication and interpersonal skills include the ability of assistant dentists to communicate well with the team, patients, and patients’ families in clinical work, with a total of six items. Teamwork refers to the ability of assistant dentists to treat patients in good cooperation with senior physician, team members, and nurses, with a total of four items. Health promotion refers to the ability of assistant dentists to take preventive measures for oral common diseases and frequently occurring diseases, reduce occupational exposure, and promote social health in the process of clinical work, with a total of five items. Professional values of doctors are the good moral character, quality, and values required by a qualified doctor, with a total of seven items. Information management ability includes the ability to apply modern information technology to promote efficient diagnosis and treatment in the workplace, with a total of six items. These explained 18.72 %, 12.31 %, 10.43 %, 10.17 %, 7.48 %, 5.29 %, and 4.01 % of total variance, respectively. Collectively, these seven factors explained 68.41 % of total variance. EFA results were statistically significant and are shown in Tables 2 and 3.

**Table 2:** Rotated component matrix

Item		Component						
		**1**	**2**	**3**	**4**	**5**	**6**	**7**
item11	Identify malocclusions and indications for implantation	0.755						
item5	General oral consciousness	0.717						
item2	Oral-disease–related examination	0.657						
item14	Simplify terms for patients	0.638						
item8	Should be under the guidance of a senior physician	0.616						
item13	Offer different treatment options	0.612						
item4	Standardize reporting of clinical problems	0.597						
item7	Identify emergency and critical patients	0.597						
item3	Regulate medical care	0.592						
item10	Operate and interpret apical projection	0.591						
item12	Refer patients in a timely manner	0.587						
item1	Document/record case history	0.574						
item9	Standardize the use of oral equipment	0.564						
item15	Knowledge of drug indications/contraindications	0.532						
item6	Reasonable diagnosis and treatment plan	0.522						
item17	Knowledge of dialect		0.778					
item16	Can study on one’s own		0.635					
item18	Basic oral knowledge		0.625					
item19	Continuous learning of oral knowledge		0.621					
item20	Focus on new technologies		0.372					
item21	Can manage children’s behavior		0.353					
item22	Listening skills and empathy			0.693				
item25	Communicate the diagnosis and treatment plan accurately and comprehensively			0.642				
item27	In-person follow-up			0.642				
item23	Good doctor–patient relationship			0.596				
item24	Protect patient privacy			0.562				
item26	Communicate with supervisors and peers			0.523				
item29	Proactive; take responsibility				0.642			
item31	Coordination with colleagues				0.627			
item28	Cooperate with and respect suggestions from colleagues				0.618			
item30	Switch between assistant and doctor roles				0.603			
item32	Show team entrepreneurship				0.325			
item34	Prevent cross-infection					0.622		
item33	Report statutory infectious diseases in a timely manner					0.605		
item37	Oral-disease prevention–related procedures					0.592		
item36	Active health education and promotion					0.585		
item35	Avoid occupational exposure					0.554		
item42	Good humanistic qualities						0.627	
item40	Avoid medical waste						0.622	
item41	Can self-evaluate						0.609	
item44	Professional dedication						0.598	
item43	Patient centered						0.592	
item38	Moral code and ethics						0.572	
item39	Integrity and preciseness						0.521	
item45	Information retrieval capability							0.775
item50	Effective use of social media							0.753
item47	Use health information systems							0.643
item46	Use information technology in health education							0.598
item48	Manage electronic medical records							0.596
item49	Have a patient management system							0.506
	Variance	18.72 %	12.31 %	10.43 %	10.17 %	7.48 %	5.29 %	4.01 %

**Table 3:** Factor analysis item partition results

Factor	Item	The content of explanation
FAC1	item1, item2, item3, item4, item5, item6, item7, item8, item9, item10, item11, item12, item13, item14, item15	clinical skills and medical care
FAC2	item16, item17, item18, item19	medical knowledge and lifelong learning
FAC3	item22, item23, item24, item25, item26, item27	communication and interpersonal skills
FAC4	item28, item29, item30, item31	teamwork
FAC5	item33, item34, item35, item36, item37	health promotion
FAC6	item38, item39, item40, item41, item42, item43, item44	professional values
FAC7	item45, item46, item47, item48, item49, item50	information management ability

### Confirmatory-factor analysis

The other half of the survey data were used for CFA, using SSPS Amos. Further CFA showed that the tested model had good model fit (Table 4). Among the many fitness indicators, *χ*^*2*^ is the most commonly reported. It can be used with degrees of freedom (df) to indicate the probability of the model’s correctness. It must be admitted that *χ*^*2*^ is very sensitive to sample size and tends to produce significant results with large samples [31]. *χ*^*2*^/df is a direct test of the similarity between the sample covariance matrix and the estimated covariance matrix. Statistically, its theoretical expected value is generally 2–3; the closer the root mean square error of approximation (RMSEA) value to 0, the better the model fit [32, 33]. Suggested sizes of standardized path coefficients are small, <0.10; medium, ~0.30; and large, >0.50 [34]. Statistics used to evaluate fit effect included RMSEA < 0.08, goodness-of-fit index (GFI) > 0.9, comparative-fit index (CFI) > 0.95 and normed-fit index (NFI) > 0.95 [25].

**Fig. 2:**
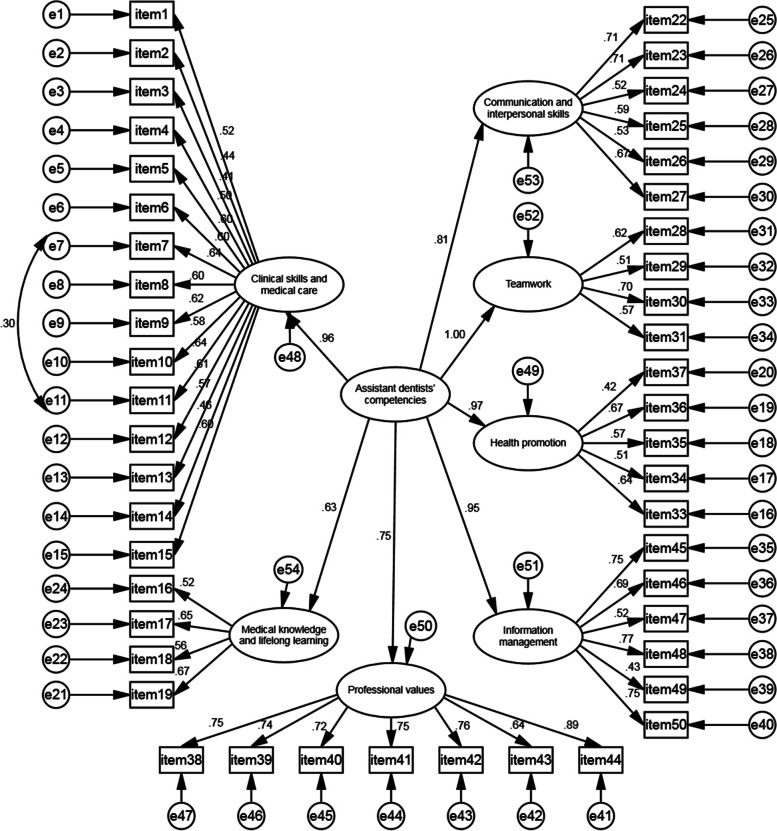
Results of confirmatory-factor analysis of general competency model for assistant dentists. Standardized coefficients and measurement errors are shown

As shown in Fig. 2, the model was adjusted according to the size of the modification indices (MIs), and a relationship was established between e7 and e11. After adjustment, the latent variable of oral assistant-physician competence was composed of seven manifest variables. Although the final model of this study was not exactly the same as the initial theoretical model of the scale, the number and structure of factors of the scale were basically unchanged, and the fit indices met all requirements with an ideal fit effect, except for some items that were deleted. Overall, it can be concluded that the model’s GFI is not merely acceptable but high quality, indicating that the overall model has good structural validity.

**Table 4:** Fitness indices for confirmatory-factor analyses of assistant-dentist competency model (n = 1286)

Index of model fit	*χ*^*2*^/df	RMSEA	NFI	CFI	GFI	IFI
**Result**	2.758	0.047	0.954	0.956	0.914	0.956

RMSEA = root mean square error of approximation; NFI = normed-fit index; CFI = comparative-fit index; GFI = goodness-of-fit index; IFI = incremental-fit index

## Discussion

### Competency of assistant dentists

This project was undertaken to construct a competency model for assistant dentists and to evaluate the reliability and validity of the model. The model we established has 7 dimensions and 47 items. It is similar to the AEDA framework (Table 5) [16, 19] in clinical skills and medical care (patient care in ADEA), medical knowledge and lifelong learning (professionalism and critical thinking in ADEA), information management ability and professional values (practice management and informatics in ADEA), communication and interpersonal skills and teamwork (communication and interpersonal skills in ADEA), and health promotion (health promotion in ADEA). Although the basic framework requirements are the same, the object of our research is assistant dentists, and the specific content is different for each ability. This is because China’s assistant dentists have a relatively flexible scope of practice. The state has set only minimal requirements for assistant dentists and permitted them a relatively wide legal scope of practice. They can adjust their scope of practice according to their medical resources. Therefore, assistant dentists resemble a combination of dental therapists/hygienists and dentists engaged in basic diagnosis and treatment.

Our findings also suggested that there are differences in competency requirements and focus between assistant dentists and dentists in China [22]. Even more than dentists, assistant dentists must accept the importance of lifelong learning ability. Assistant dentists place more emphasis on medical knowledge and lifelong learning. The National Dental Licensing Examination of China is the only means of qualifying assistant dentists, who can take dental exams after working for a few years. Delphi experts generally believe that the education and continuing education of assistant dentists after graduation is very important and that they need to attain the knowledge level of dentists through continuous learning. Although some assistant dentists work like dentists and practice independently, they are focused on service and practice and make no requirement on their scientific research ability. In general, both roles require mastery of basic theoretical knowledge of stomatology in oral expertise, basic medicine and clinical medicine; as well as the main technical skills and clinical thinking ability needed for oral clinical work. The difference is that the medical knowledge of assistant dentists is more basic and elementary; more emphasis is placed on humanistic care, good communication, teamwork and awareness of dental assistants. The skill set of assistant dentists should focus on their ability to flexibly change roles in their teams, handle relationships with team members and execute the clinical-treatment decisions of senior dentists.

Cultivating PCDs can alleviate the problem of a lack of dental staff. The presence of assistant physicians can help alleviate the shortage of medical resources in China. They have effectively undertaken the primary and secondary prevention work in the fight against COVID-19. The existence of this profession is significant for the development of healthcare in China and even worldwide. Though increasing the number of physicians is important, ensuring the quality of the oral team is even more so. Therefore, the introduction of competency models for assistant dentists has important practical significance for further strengthening the primary-level dental team. Competency is a key concept in human-resource management development and is pursued through four channels: education, training, development, and learning [35, 36]. In order to have competent human resources, education, especially medical education, should be developed in accordance with competency models and standards. Competency assessments should be carried out at every stage of the process, and a system to cultivate medical personnel should be constructed that connects the three stages of college, postgraduate and continuing education. The assistant-dentist competency model will provide basic data for the development of training programs to improve the quality of supplemental dental personnel. We are the first to put forward a job competency model for this group, which is also of indispensable significance for the training and assessment of primary-level oral-hygiene personnel in China.

**Table 5:** Comparisons of competency frameworks

Competency framework for assistant dentists of China	ADEA	ADEE
λ Clinical skills and medical care	λ Patient care	λ Diagnosis and treatment planning
λ Medical knowledge and lifelong learning	λ Professionalism	λ Clinical information gathering
	λ Critical thinking	
λ Information management ability	λ Practice management and informatics	λ Knowledge base, information, and information literacy
λ Professional values		λ Professional attitude and behavior
λ Communication and interpersonal skills	λ Communication and interpersonal skills	λ Ethics and jurisprudence
λ Teamwork		λ Therapy: establishing and maintaining oral health
λ Health promotion	λ Health promotion	λ Prevention and health promotion

### Role of assistant dentists

Assistant dentists practice independently, mainly in western China and in rural areas. Their role in China is not quite the same as that of dental therapists. In high-end dental clinics and specialized oral hospitals in China’s eastern coastal areas, assistant dentists mainly work in a fashion similar to that of dental therapists and hygienists in Europe, Netherlands, the United Kingdom (UK), and Alaska [37–40]. The main tasks include oral health promotion, prevention of oral diseases, simple dental treatments carried out in cooperation with dentists such as cleanings or taking dental impressions, and referrals for patients in need of more complex treatment. The scope of practice for assistant dentists in eastern China is analogous to a combination of the scopes of practice for old-curriculum dental hygienists (more preventive and periodontal tasks) and new-curriculum ones (more caries-related diagnostic and treatment tasks) in the Netherlands [12]. However, in less-developed areas, the role of assistant dentist has further expanded into one similar to that of a dentist. Due to the shortage of doctors in underdeveloped areas, assistant physicians in these areas are responsible for diagnosing and treating common dental issues, as well as diagnosing serious diseases and then referring patients to specialists. The scope of their practice includes treating caries, extracting teeth, creating dental prostheses, performing root canals and other work. The state has set only minimal requirements for assistant dentists and permitted them a relatively wide legal scope of practice.

It is undeniable that assistant physicians have made significant contributions to health services in China. For instance, China cannot effectively control coronavirus disease 2019 (COVID-19) without a large number of such PCDs. By 2019, China had 245,000 stomatologists, 50,000 of whom were assistant dental practitioners, which is 20.41 % of the total number of stomatologists [41]. Assistant dentists are now well established as an essential part of the primary-level dental team in the past, present and even long-term future. The same is true of oral therapists, who are part of the dental team in many countries [42]. An assistant dentist is like a general practitioner who specializes in oral medicine. The current success of New Zealand has demonstrated the superiority and effectiveness of using school dental nurses [43, 44]. Training programs for dental therapists/hygienists have been added or considered in countries/regions such as the UK, Northern Europe, and the US [45–47]. The assistant-physician model in China, the largest developing country and the world’s most populous, provides experience for other countries suffering a shortage of doctors. Particularly in LMICs, training a more community-oriented oral-health workforce rather than dentists is a practical solution to address acute workforce shortages and challenges [48].

As for other professional fields, discussions of scope of practice and independence for different groups such as dental therapists, dental hygienists, dental assistants/nurses and dental technicians are often complex [5]. Therefore, the construction of a competency model is particularly important. Job requirements should meet the organization’s developmental needs, and job responsibilities and tasks should be structured accordingly. Training programs and talent cultivation should be tailored to job requirements.

## Conclusions

The assistant-dentist competency model, which has significant levels of reliability and validity, is composed of 7 dimensions with 47 items: (a) clinical skills and medical care, (b) medical knowledge and lifelong learning, (c) communication and interpersonal skills, (d) teamwork, (e) health promotion, (f) professional values and (g) information management ability. This model is consistent with the current situation in China and has distinct Chinese characteristics. This model has been approved by the relevant agencies in China and will be applied to the goals and requirements of vocational-college education, the qualification examination outline for assistant dentists, and human-resource management of dental clinics or hospitals.

## Data Availability

All data generated or analyzed during this study are included in this published article.
